# E6 and E7 gene silencing results in decreased methylation of tumor suppressor genes and induces phenotype transformation of human cervical carcinoma cell lines

**DOI:** 10.18632/oncotarget.4525

**Published:** 2015-06-19

**Authors:** Liming Li, Cui Xu, Jia Long, Danbei Shen, Wuqing Zhou, Qiyan Zhou, Jia Yang, Mingjun Jiang

**Affiliations:** ^1^ Jiangsu Key Laboratory of Molecular Biology for Skin Diseases and STIs, Institute of Dermatology, Chinese Academy of Medical Sciences and Peking Union Medical College, Nanjing, China

**Keywords:** HPV16 E6 and E7, DNA methylation, immunoblot, cell viability and apoptosis, human cervical carcinoma cell lines

## Abstract

In SiHa and CaSki cells, E6 and E7-targeting shRNA specifically and effectively knocked down human papillomavirus (HPV) 16 E6 and E7 at the transcriptional level, reduced the E6 and E7 mRNA levels by more than 80% compared with control cells that expressed a scrambled-sequence shRNA. E6 and E7 repression resulted in down-regulation of DNA methyltransferase mRNA and protein expression, decreased DNA methylation and increased mRNA expression levels of tumor suppressor genes, induced a certain apoptosis and inhibited proliferation in E6 and E7 shRNA-infected SiHa and CaSki cells compared with the uninfected cells. Repression of E6 and E7 oncogenes resulted in restoration of DNA methyltransferase suppressor pathways and induced apoptosis in HPV16-positive cervical carcinoma cell lines. Our findings suggest that the potential carcinogenic mechanism of HPV16 through influencing DNA methylation pathway to activate the development of cervical cancer exist, and maybe as a candidate therapeutic strategy for cervical and other HPV-associated cancers.

## INTRODUCTION

Cervical cancer remains the third most common cancer in women worldwide [1] and the leading malignancy in developing countries, accounting for 15% of whole female cancer cases [2, 3]. Although well organized screening and early therapeutic schedule have been carried out, the occurrence of invasive cervical cancer is still common, especially in developing areas. It is well known that almost all cases of cervical carcinoma are initiated by infection with high-risk HPVs [4].

HPV is a non-enveloped double-stranded DNA virus that naturally infects skin and mucosal epithelia. HPV is responsible for approximately half a million cases of cervical cancer in the world each year [5]. A group of HPVs including HPV-16, HPV-18, HPV-31, HPV-33, HPV-45, and others has been designated as “high-risk,” and persistent infection of the cervix with these high-risk HPVs is considered to be the necessary cause of cervical cancer [6, 7]. Numerous lines of epidemiological and molecular pathology evidence reveal that HPV16 is most prevalent and accounts for 65.2% of all genotypes in cervical cancers [8]. High-risk HPVs encode E5, E6, and E7 proteins, the constant expression of which is needed for a cell to remain malignant [9].

There are currently no specific antiviral compounds that are active against HPV. RNA interference has recently been recognized as a powerful and promising tool to inhibit specific gene expression through endonucleasic cleavaging and degradating of homologous mRNA stably [10]. RNAi shows great potential for the treatment of viral diseases, genetic disorders, and cancer [11-15]. The shRNA strategy has already been tried in the context of HIV-1 treatment and shown to successfully overcome viral escape and mutation [16-20]. To date, various *in vitro* and *in vivo* studies have demonstrated that silencing HPV16 E6/E7, with siRNA or shRNA, leads to cervical cancer cells undergoing apoptosis or senescence, growth inhibition [21-24] and inhibition of tumor growth *in vivo* [25-27]. However, using shRNAs to target purpose genes has not been previously investigated for cervical cancer by changing the epigenetics and is all the more important as this may be a new method for cervical cancer diagnosis.

Meanwhile, some studies have suggested that aberrant DNA methylation in cancer have been considered to have great potential as a source of locus-specific biomarkers that can be used in early detection and monitoring of cancer [28, 29]. It was reported that MT1G, NMES1, RRAD, SFRP1, SPARC and TFPI2 were more often methylated in invasive cervical cancer samples than in normal ones, suggesting that these 6 genes may be potential tumor suppressor candidate for cervical cancer [30]. And then, several studies recently demonstrated the siRNA-induced transcriptional gene silencing is regarded to be associated with epigenetic alterations including DNA methylation, histone modification, and chromatin conformation [25-27]. Methylation-specific high resolution melting (MS-HRM) [31] is capable of analyzing homogeneous methylation in a semiquantitative manner [32]. Estimation of methylation levels in MS-HRM is performed on the basis of a comparison of melting profiles of screened samples and standards of known ratios of methylated and unmethylated DNA [33]. On the other hand, Burgers et a l. found HPV 16 E7 can associate *in vitro* and *in vivo* with the DNA methyltransferase DNMT1, and stimulate the methyltransferase activity of DNMT1 *in vitro* [34]. Leonard and Au Yeung et al reported that HPV16 can upregulate the expression of DNMT1 and DNMT3B [35, 36].

Based on the above description, suppression of HPV16 E6 and E7 by RNAi will reduce the methylation of tumor suppressor genes, leads to cervical cancer cells undergoing apoptosis, senescence, growth inhibition. This RNAi therapy may prove to be valuable for the treatment of cervical cancer. However, experimental evidence showing that HPV16 is causally associated with the methylation of 6 suppressor genes in cervical carcinomas cells is lacking. Experimental data showing that transcriptionally active HPV16 is required for malignant transformation of HPV16-positive human cervical carcinoma cell lines through the methylation of 6 suppressor genes have not been clearly elucidated. we need further evidence to elucidate the potential mechanisms led by HPV 16 via epigenetic pathway.

To investigate the above issue and the potential role for shRNA in the treatment of cervical cancer, we used the shRNA delivered by a lentivirus will effectively, specifically and stably suppress HPV 16 E6 and E7 expression. The aim of this study is to assess whether E6 and E7 gene silencing results in decrease methylation of tumor suppressor genes and induces phenotype transformation of human cervical carcinoma cell lines.

## RESULTS

### Choice of shRNA sequences for viral gene silencing

**Figure 1:**
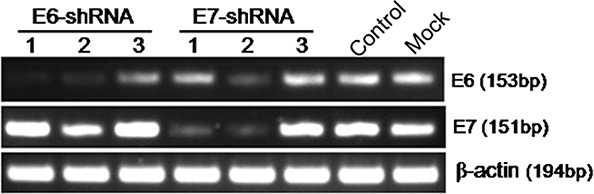
Effect of E6 and E7 shRNAs on HPV16 E6 and E7 mRNA expression by RT-PCR Untreatment (Mock) SiHa cells were transfected with E6-shRNA-1/2/3, E7-shRNA-1/2/3 and control shRNA. After 48 h of incubation, mRNA was extracted and reverse transcripted. cDNA was PCR-amplified with E6 and E7 gene-specific primers and β-actin primers as control.

To disrupt HPV16 E6 and E7 gene expression, we designed 6 shRNA oligonucleotides that targeted different locations within the HPV16 E6 or E7 transcript (Supplementary Fig. 1). We cloned these oligonucleotides into a shRNA expression vector, and the shRNA constructs were used to transiently transfect HPV16-positive cervical carcinoma cell lines (SiHa) with Lipofectamine. Two days after transfection, we used RT-PCR to analyze the expression of HPV16 E6 and E7 mRNA. The scrambled shRNA had no effect on E6 and E7 mRNA levels compared with uninfected cells. Infection with constructs expressing two shRNAs (E6-shRNA-1/2 and E7-shRNA-1/2) showed the dramatic silencing efficiency compared with uninfected cells (Fig. 1). But E6-shRNA-2 and E7-shRNA-2 revealed poor specificity. So we used the E6-shRNA-1 and E7-shRNA-1 as E6-shRNA and E7-shRNA for subsequent experiments.

### Effects on expression of 12 genes by lentivious-shRNA targeting HPV16 E6 and E7 genes in HPV16-positive cervical carcinoma cells

These lentiviruses expressing E6-, E7-, and control shRNA were used to infect HPV16-positive cervical carcinoma cell lines (SiHa and CaSki). At every 24 hours within 4 days after lentiviruses infection of SiHa and CaSki, we used Q-PCR to analyze the expression of E6, E7, DNA methyltransferase genes (DNMT1, DNMT3A, DNMT3B and DNMT3L) [37], and tumor suppressor genes (MT1G, NMES1, RRAD, SFRP1, SPARC and TFPI2).

**Figure 2:**
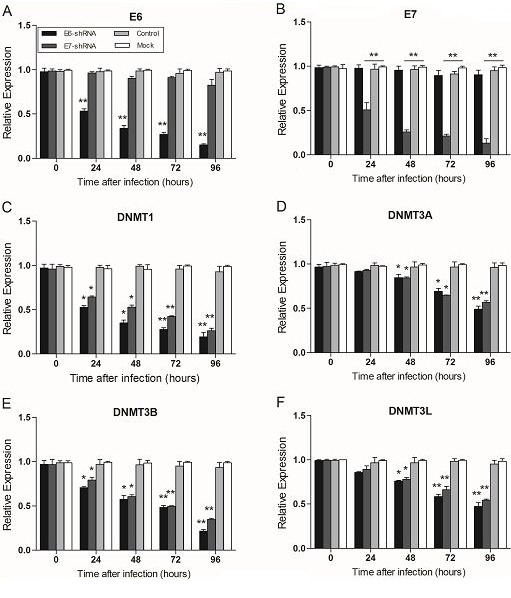

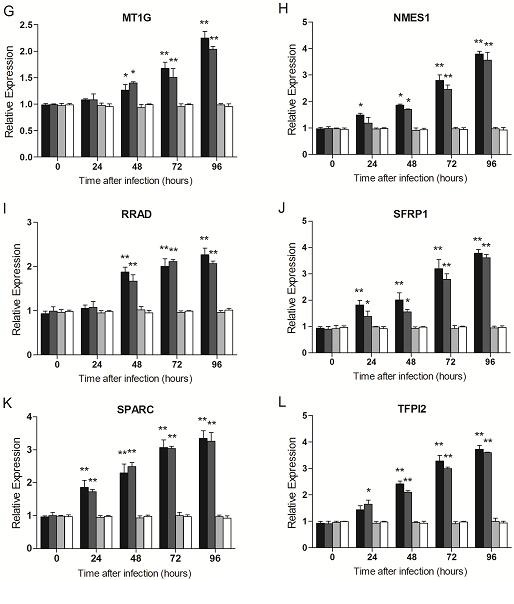
Effect of E6- and E7- shRNAs expression on E6, E7, tumor suppressor genes and DNA methyltransferase genes of SiHa cells RNA was harvested at 0, 24, 48, 72, 96 hours after lentiviruses infection of SiHa and CaSki (data not shown) cells and subjected to gene expression analysis by Q-PCR normalized to β-actin expression. Each column represents at least 3 repeats. For all genes analyzed, the significant difference of the E6 or E7 shRNA-infected cells (SiHa and CaSki) was compared with the corresponding uninfected cells (Mock) at the each time point. *p < 0.05, **p< 0.001.

In SiHa, the expression of E6 and E7 was decreased remarkablely over time after infection with lentiviruses expressing two shRNAs (E6-shRNA and E7-shRNA). The infection caused an approximately 50% knockdown of HPV16 E6 and E7 mRNA levels respectively compared with uninfected cells (*p*<0.01) at 24 h, and more than 80% at 96 h (*p*<0.01) (Fig. 2A and 2B). At the same time, expression of all the other 9 genes at mRNA levels had Significant change compared with uninfected cells after 48 h after infection (*p*<0.01) (Fig. 2C-2L). However, when SiHa (Fig. 2) and CaSki (data not shown) cells were infected with the control lentivirus expressing control shRNA, the expression of every mRNA was similar to their expression in uninfected cells (*p*>0.05).

### Effects of E6- and E7- shRNA on the methylation of tumor suppressor genes

**Figure 3:**
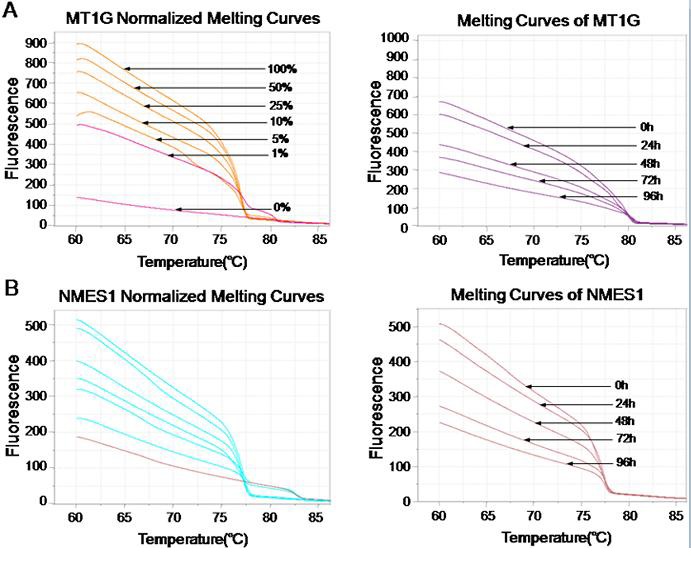

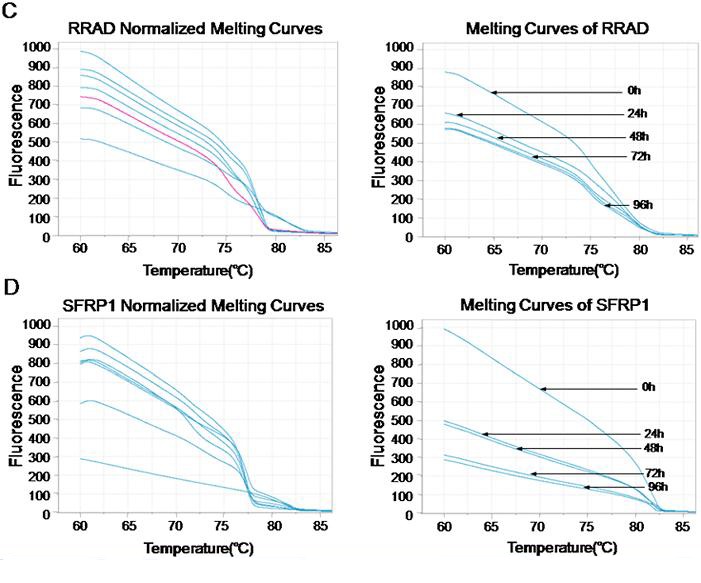

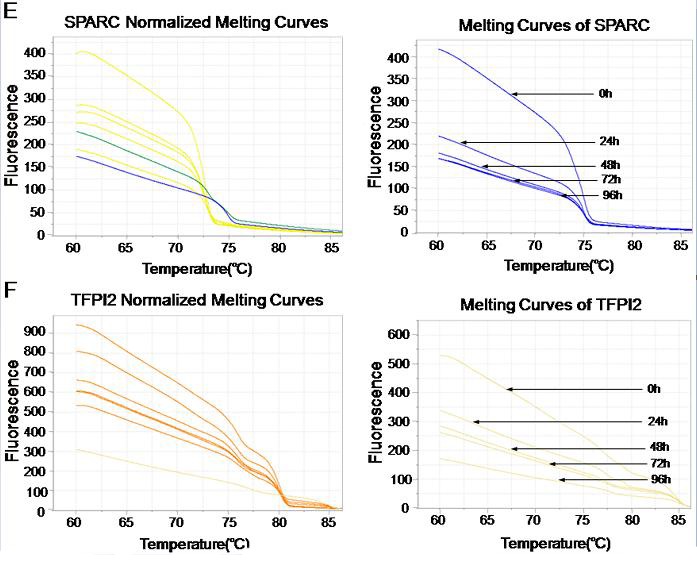
Effect of E6- and E7- shRNAs on DNA methylation of tumor suppressor genes by MS-HRM analysis using serial dilutions of methylated DNA (from 100% to 0%) as a template of SiHa cells Melting curves of SPARC (**A**), TFPI2 (**B**), RRAD (**C**), SFRP1 (**D**), MT1G (**E**) and NMES1 (**F**) genes were showed above. Similar results were obtained for CaSki cells.

MS-HRM was previously described as a specific method for methylation analysis [38]. It relies upon the precise monitoring of the change of fluorescence intensity as a DNA duplex melts. To determine the DNA methylation of the 6 tumor suppressors (MT1G, NMES1, RRAD, SFRP1, SPARC and TFPI2) after SiHa and CaSki cells infected with lentiviruses that expressed E6-, E7- and control shRNA, a standard dilution series was used with different ratios of methylated-to-unmethylated template (0, 1, 5, 10, 25, 50, and 100% methylated). All the 6 genes MS-HRM assays were able to stably detect 1% methylated DNA in a background of unmethylated DNA, while there was no significant distinction of Normalized Melting Curves between 5-25% of SFRP1 methylation range, and 5-10% of TFPI2 methylation range (Fig. 3D and 3F). In the analysis, we found that methylation of 6 genes were significantly decreased after SiHa cells infected with lentiviruses that expressed E6 (Fig. 3), and E7 (data not shown), Similar results were obtained from CaSki cells (data not shown).

### Effects of E6- and E7- shRNA on the expression of DNA methyltransferase protein

**Figure 4:**
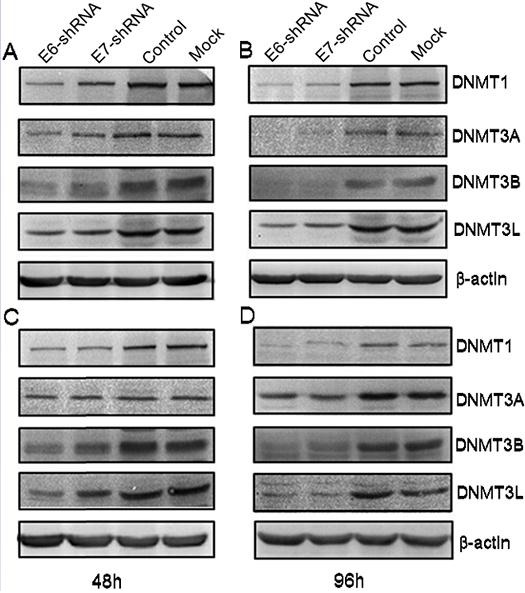
Effect of shRNA-mediated repression of HPV16 E6 and E7 on DNA methyltransferase expression Immunoblot analysis of DNMT1, DNMT3A, DNMT3B and DNMT3L protein levels in SiHa (**A** and B) and in CaSki (**C** and **D**) were shown above. Protein extracts were made from uninfected (Mock) CaSki and SiHa cells, and from cells infected for 48 and 96 h with lentivirus expressing E6-, E7- and control- shRNA. Extracted protein samples (50μg per lane) were subjected to electrophoresis and immunoblotting with antibodies specific for the 4 protein and β-actin as control for equal loading.

To assess the effect of E6 and E7 gene silencing on the protein expression levels of the DNA methyltransferase, we examined the expression of these 4 proteins at 48 and 96 h after lentivirus infection using antibodies specific for DNMT1, DNMT3A, DNMT3B and DNMT3L by immunoblotting. Consistent with the results of E6 and E7 mRNA expression obtained by Q-PCR, immunoblot analysis revealed a marked decrease in the level of protein in E6 and E7 shRNA-infected SiHa and CaSki cells compared with uninfected or control shRNA-infected SiHa and CaSki cell lines (Fig. 4).

### Phenotypic effects of HPV16 E6- and E7- shRNA in SiHa and CaSki cells/

We observed substantial morphological changes in CaSki cell infected with E6 and E7-shRNA for 48 h, compared with cells infected with the control shRNA or uninfected cells. The E6- and E7- shRNA-infected cells shriveled, detached from the plate, and died (data not shown). Similar morphological changes, as well as cell death, were also observed in E6- and E7- shRNA-infected SiHa cells, but to a little lesser extent (data not shown).

**Figure 5:**
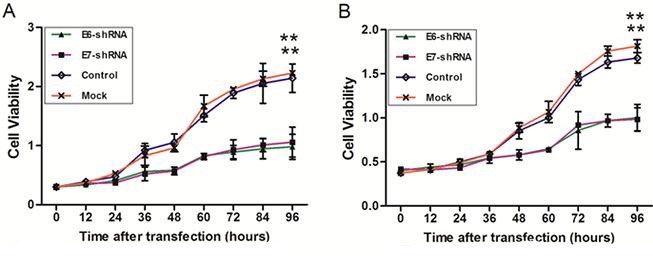
Effect of E6 and E7 repression on CaSki and SiHa cell viability SiHa (**A**) and CaSki (**B**) cells were infected with lentiviruses expressing E6-, E7- and control- shRNA in a 96-well plate. Cell viability was measured with the use of the CCK-8 assay at 9 different time points after infection. The change of cell viability was determined by measuring the mean optical density at 450 nm compared with uninfected (Mock) cells for each cell line. *p < 0.05, **p< 0.001.

To verify whether inhibition of E6 and E7 expression could induce a reduction in cell viability at different time points (0, 12, 24, 36, 48, 60, 72, 84 and 96 h) after infection, CCK-8 assay was used in E6- and E7- shRNA-infected SiHa and CaSki cells. After 96 h of culture, the cellular proliferation was reduced by 56.1% in CaSki cells transduced by E6-shRNA and 52.5% in cells transduced by the E7-shRNA compared with uninfected cells (both *P*<0.01). At the same time, the cellular proliferation was reduced by 44.8% in SiHa cells transduced by the E6-shRNA and 45.8% in transduced by E7-shRNA compared with the uninfected cells (both *P*<0.01) (Fig. 5). No significant differences existed between the control and uninfected cell groups in both SiHa and CaSki cells (Fig. 5).

**Figure 6:**
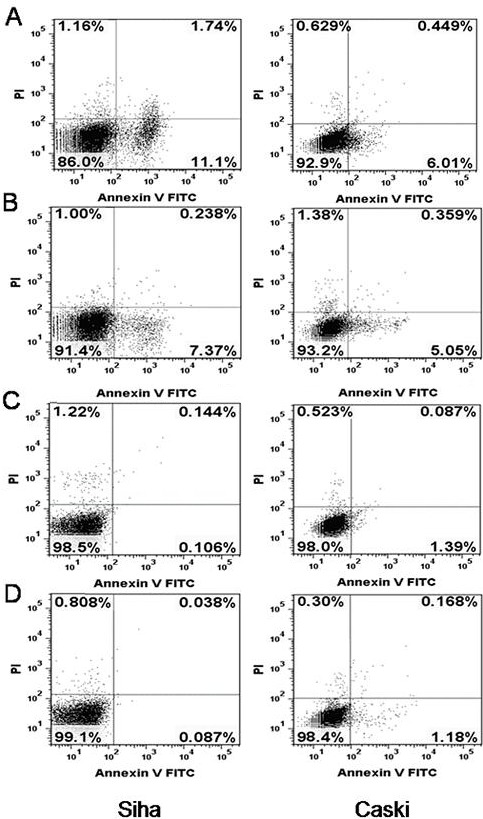
Effect of HPV16 E6 and E7 repression on apoptosis (**A**) SiHa and CaSki cells infected with lentiviruses expressing E6-shRNA. (**B**) SiHa and CaSki cells infected with lentiviruses expressing E7-shRNA. (**C**) SiHa and CaSki cells infected with lentiviruses expressing control shRNA. (**D**) Uninfected SiHa and CaSki cells. Cells were assayed by flow cytometry for annexin V binding and PI staining 72 hours after infection. The logarithm of annexin V-fluorescein isothiocyanate fluorescence and the logarithm of PI fluorescence were plotted on the *x* and *y* axes of the cytogram. The lower right quadrant shows the percentage of viable cells in the early stages of apoptosis (ie, annexin V-positive, PI-negative cells), the upper right quadrant shows the percentage of cells in a later stage of apoptosis or necrosis (annexin V-positive, PI-positive cells), the lower left shows the percentage of cells in the proliferating-senescence state and the upper left quadrant shows the percentage of dead cells.

Data from previous studies showed that repression of HPV16 E6 and E7 oncogenes in cervical cancer cells results in apoptosis [32]. We examined whether the loss of cell viability that we observed in E6- and E7- shRNA-infected SiHa and CaSki cells was caused via apoptosis by using flow cytometry to assess annexin V binding and PI permeability of nonpermeabilized SiHa and CaSki cells 72 h after infection with lentivirus expressing the E6, E7 and control shRNA (Fig. 6). Generally, the apoptotic rates (upper and lower right quadrants combined) were relatively low. The apoptotic rate was 12.8, 7.6, 0.25 and 0.13% for E6-, E7-, control shRNA-infected and uninfected CaSki cells; meanwhile, 6.5, 5.4, 1.5 and 1.4% for E6-, E7-, control shRNA-infected and uninfected SiHa cells, respectively. However, the apoptosis of E6- and E7- shRNA-infected CaSki cells were more than that in the blank control (both *P*<0.01), this phenomenon is also present in the SiHa cells (both *P*<0.05).

## DISCUSSION

The lentiviral vector has been explored as a vehicle for stable and durable gene therapy in preclinical treatment and clinical trials [39, 40]. In addition, shRNA itself may be developed as a novel anti-viral agent to counter viral infection and disease [11-15], such as cervical cancer [25-27]. Therefore, we hoped it could do so for lentiviruses that expressed the various shRNAs in cervical cancer cells.

In this study, we investigated the efficacy of HPV16 E6- and E7- shRNA in silencing E6 and E7, respectively. A dramatic decrease of E6 and E7 mRNA was observed with E6- and E7-shRNA infection, in agreement with previous studies [21]. And shRNA-mediated repression of E6 and E7 resulted in activation of the tumour suppressor genes through reducing their methylation level and induction of apoptosis in HPV16 positive cervical carcinoma cell lines. These results suggest that continuous expression of HPV16 E6 and E7 with high methylation of tumour suppressors in HPV16 positive human cervical cancer cells might be required to maintain the malignant phenotype and actively prevent the execution of apoptosis.

The methylation status of multiple tumor suppressor genes may serve as biomarkers for early cancer diagnostics [28-30], for prediction of prognosis and for prediction of response to treatment. Therefore, it is important that methodologies for detection of DNA methylation continue to evolve. Establishing a link between cervical cancer and the methylation of tumour suppressors after HPV16 infection is very interesting. As shown by Laird and Levenson, the DNA methylation can be used in early detection and monitoring of cancer as a source of locus-specific biomarkers [28, 29]. At the same time, previous studies demonstrated the siRNA-induced transcriptional gene silencing is regarded to be associated with epigenetic alterations including DNA methylation [25-27]. HPV16 DNA positive cervical tumors bearing the high methylation tumour suppressors have also been described [30]. However, detection of HPV16 and the methylation of tumour suppressors in cervical cancer cells per se does not prove a causal association. Herein we present the first comprehensive quantitative analysis of the methylation status of 6 tumour suppressor genes in cervical cancer cells. Our results suggest that shRNA-mediated repression of E6 and E7 resulted in reducing the methylation of tumour suppressors and the expression of DNMTS. The results suggest that 6 tumour suppressors may be a target for aberrant DNA methylation in cervical cancer cell lines. In conclusion, HPV lentiviruses encoding shRNA may provide another means of cervical cancer therapy, and using lentiviruses seems to be a more promising.

Many different PCR-based methods for the detection of DNA methylation have been developed [41]. Methylation specific PCR [42] is the most widely used as it is very sensitive, cost-effective, and does not require specialized equipment. However, MSP is a non-quantitative method [43] and prone to false-positive results [44-46]. In a technique called MethyLight [47], the use of TaqMan probes allow for methylation levels to be estimated. This, however, increases the cost of the experiment. MS-HRM analysis has been considered as the most rapid and sensitive method to assess the presence of DNA methylation. The principle of this method is that PCR products generated from bisulfie-treated DNA templates with different contents of methylcytosine show differences in melting temperature, which can be resolved by melting analysis in a thermal cycler coupled with a fluorometer [32]. Quantifiation was carried out by interpolation on a standard curve generated with serial dilutions of methylated and unmethylated DNA. It can detect even 0.1-1% of DNA methylation in an unmethylated background, minimizing possible sample contamination, and requiring only low amounts of DNA template [48]. This method can also clearly show the change trend of gene methylation over time.

In our experimental system, repression of E6 and E7 oncogenes led to degradation of DNMTS. In addition, in SiHa and CaSki cells, Knockdown of E6 led to the repression of DNMT1 protein [36]. Burgers et al. [34, 36] showed that HPV 16 E7 associate *in vitro* and *in vivo* with the DNA methyltransferase DNMT1 and E7 can stimulate the methyltransferase activity of DNMT1 *in vitro*. Upregulation of the DNMT1 and DNMT3B was found at primary human foreskin keratinocytes after transfection with episomal forms of high-risk HPV types had also been described [35]. However, the specific factors that determine the cellular response to E6 and E7 repression remain unclear. The pattern of gene expression in response to the repression of E6 and E7 genes as well as the inactivation of tumor suppressors that we observed in HPV16-positive cervical cancer cell lines was consistent with the observed apoptotic cellular response because several of the genes whose expression was altered and effected the cell proliferation.

A unique feature of HPV-associated cancer is that the maintenance of the malignant aspect, just as apoptosis resistance, which allows them to proliferate continuously and survive immortally under the abnormal growth stimuli [49]. In some virus-associated tumors, antiapoptotic activities are usually derived from viral proteins [50]. Various studies [21, 25] have reported that downregulation of both E6 and E7 expression by E6 or/and E7 siRNA, through the post-transcriptional level, leads to retarded growth of HPV16- positive cervical cancer cell lines. In our study, the decreases in E6, E7 and methylation of the tumour suppressors also partially altered the malignant characteristics. At the same time, cells treated by the shRNA targeting E6 or E7 represented a remarkably decreased proliferation, as well as changed cellular growth morphology, which may attribute to increased expression of tumour suppressors as well as decreased the methylation of the expression of them in HPV16 induced cervical cancer cells.

A number of issues have to be taken into account when interpreting the results of the present study. First, the weakness of this study is that it focuses in a group of tumor suppressor genes that have been shown methylated in HPV-positive cervical carcinoma by previous studies [31] and it is not expanded on novel tumor suppressor genes. However, our aim was to find and provide the evidence about the relationship between HPV 16 and tumor suppressor genes through associated DNA methylation pathway, so we chose the six genes published and demonstrated already. Second, the more interesting question would be whether E6 and E7 gene silencing are pathogenically involved in the development of human cervical carcinoma. The *in vitro* design severely limits the interpretability of the data. Further work should be considered.

Our findings support the notion that cervical cancers that contain transcriptionally active HPV16: inactivation of tumors suppressor pathways occurs via degradation of the methylation of the 6 genes, respectively, and repression of these viral oncogenes restores these tumor suppressor pathways and leads to apoptosis. Thus, this study showing that continuous expression of HPV16 E6 and E7 oncogenes will cause the inactivation of the 6 tumor suppressor pathways and the maintenance of the proliferative state of HPV16-positive cervical carcinoma cancer cells. In summary, the ability to suppress the methylation of tumour suppressor using HPV16 E6- and E7- shRNA opens new and exciting routes to the understanding of mammalian cell biology and its pathology. These results could partly contribute to the establishment of a causal association between HPV and cervical cancer. Validation of a causal relationship, if followed by the rapid deployment of preventive and therapeutic strategies, such as anti-HPV vaccines, could have a major impact on public health.

## MATERIALS AND METHODS

### Cell lines and culture conditions

The human cervical carcinoma cell lines SiHa and CaSki contain integrated HPV-16 genome, about 600 copies (CaSki) and one to two copies (SiHa) [51]. These two cell lines and human embryonic kidney 293T cells were obtained from our laboratory and maintained in Dulbecco's modified Eagle medium (DMEM) containing 10% fetal bovine serum (Gibco, Gaithersburg, MD), 2 mM L-glutamine, and 100 U/mL penicillin-streptomycin (PBS) in a humidified (37°C, 5% CO_2_) incubator, grown in 25-cm^2^ culture flasks, and passaged when they reached 80% confluence.

### Design and production of plasmids expressing E6 and E7 specific shRNA

All shRNA sequences targeting the HPV16 E6 and E7 transcripts were designed according to published criteria [52] by using the BLOCK-iT RNAi Designer algorithm (Invitrogen, Carlsbad, CA) and were screened against the human genome by using a BLAST search (www.ncbi.nlm.nih.gov/bl/blast) to assess sequence specificity and avoid unintentional silencing of host cell genes. We selected six different target sites within the transcribed HPV16 E6 and E7 mRNAs according to the Invitrogen Web-based guidelines (https://rnaidesigner.classic.invitrogen.com/rnaiexpress/index.jsp) (Supplementary Table 1). Complementary oligonucleotides encoding shRNAs that targeted each of these six sites were synthesized, annealed, and cloned into the the pENTR/U6 vector (Invitrogen), resulting in lentiviral plasmids E6-shRNA-1/2/3, E7-shRNA-1/2/3. For a negative control shRNA, we cloned a scrambled-sequence oligonucleotide into pENTR/U6 vector, resulting in control shRNA. The sequence was submitted to a BLAST search against the human genome sequence to ensure that no gene of the human genome was targeted. The resulting plasmids were purified by using a Plasmid Miniprep Kit (Omega, Georgia, GA) according to the manufacturer's protocol, and the presence of the correct inserts was confirmed by DNA sequencing.

### Screening of shRNAs-transient transfection and RT-PCR

Three groups were included in the experiments: the shRNA transfected with liposome was considered as the experiment group; scrambled shRNA transfected with lipofectamin 2000 (Invitrogen) was considered as control and cells without treatment as blank control (Mock) [53]. The day before transfection, the SiHa cells were trypsinized and seeded into 6-well plates without antibiotics, 1.5×10^5^ cells per well. While growing to 70–80 % confluency, cells were transfected with 100 pmol each shRNA plasmid using lipofectamin2000 according to manufacturer's instructions. About 48 h after transfection, cells were washed once with PBS and harvested for total RNA extraction, RNA isolation was completed using TRIzol® reagent (Invitrogen) as instructed by the manufacturer. Single-stranded complementary DNA (cDNA) was synthesized from 1 μg of total RNA in a 20 μL volume by using a RevertAid First Strand cDNA Synthesis Kit (Thermo Scientific, Rockford, IL). The cDNA samples were diluted and subjected to PCR amplification.

In order to find out the most suitable shRNAs, primers (Supplementary Table 2) were designed for RT-PCR based on the β-actin, HPV16 E6 and E7 sequences. MFOLD (http://frontend.bioinfo.rpi.edu/Applications/mfold/cgi-bin/dna-form1.cgi) and BLAST were used to check primers’ specificity. RT-PCR were performed with a total volume of 20 μL in each tube, each containing 2 μL 10×buffer (Mg^2+^ plus), 1 μL cDNA, 0.25 μM of each primer, 0.25 μM of each dNTP and 2.0 U Taq DNA polymerase (Takara, Dalian, China). Conditions for amplification were 5 min at 95°C followed by 27 cycles of 95°C for 30 s, 60°C for 30 s, 72°C for 10 s and 3 final extension of 72°C for 10 min. The values of β-actin mRNA were used as an endogenous control to normalize for differences in the amount of total RNA.

Amplified products were separated on a 1.5% agarose gel containing ethidium bromide. Intensities of E6 and E7 bands with different shRNA tranfected were observed with a UV transilluminator (wavelength = 300 nm). And then, PCR-amplified fragments were sequenced by Beijing Genomics Institute (BGI) for correct squences of E6 and E7. To confirm the result, all transfection data are representative of 3 repeat independent transfections using at least two independent preparations of both DNA and plasmid clones. According to the result, the best shRNA was selected and used as E6-, E7-, and control shRNA for further analysis.

### Production of lentivirus expressing selected shRNA

Lentivirus production was done using the BLOCK-iT lentiviral RNA interference expression system (Invitrogen). Briefly, the recombination reaction between the pENTR/U6 plasmid encoding shRNA (E6, E7 and control) and plenti6/BLOCK-iT DEST was done to generate the plenti6/BLOCK-iT-DEST expression construct. To generate recombinant lentiviruses that expressed the various shRNAs, 2 × 10^6^ 293T cells were seeded on 100-mm plates and incubated overnight at 37°C. The 293T cells were cotransfected with 9 μg of the ViraPower Packaging Mix and 3 μg of plenti6/BLOCK-iT-DEST expression plasmid DNA using Lipofectamine 2000 [54]. After 6-8 h, the medium was changed and substituted with serum medium. The virus containing cell culture supernatants were collected at 48 h and 72 h post transfection, concentrated by centrifugation and passed through 0.22 μ m (pore size) membrane filters (Millipore, Billerica, MA) to be used for infection of cervical carcinoma cell lines. The viral stocks were separated into aliquots and stored at −80°C and would infected cells with the same volume DMEM.

### Q-PCR

In order to explore the dynamic change of the E6, E7, DNA methyltransferase genes (DNMT1, DNMT3A, DNMT3B and DNMT3L), and tumor suppressor genes (MT1G, NMES1, RRAD, SFRP1, SPARC and TFPI2) at different time (24, 48, 72, 96 h) after SiHa and CaSki cells uninfected and infected with lentiviruses that expressed E6-, E7- and control shRNA, total RNA was isolated and reverse transcripted into cDNA. And then, Q-PCR was carried out using the QuantiFast SYBR Green PCR Kit (Qiagen, Valencia, CA) according to the manufacturer's instructions. The 13 pair primers (Supplementary Table 2) were designed by primer 5, and sequences were BLAST-confirmed for specificity. Briefly, 1 μL of the diluted cDNA sample was added to a tube containing 10 μL 2×SYBR Green Master Mix, 0.05 μM primer pair, and 8 μL RNase-free water.

The Q-PCR was run on ABI 7300 (Applied Biosystems, Rockford, IL). Each sample was run in triplicate. The condition for Q-PCR was as follows: 50°C for 2 min and 95°C for 5 min followed by 40 cycles of 95°C for 20 s, 60°C for 30 s and. The condition for last stage for the dissociation curve was as follows: 95°C for 15 s, 60°C for 1 min and 95°C for 15 s [55].

Q-PCR amplification data were analyzed and threshold cycle (Ct) numbers were automatically determined by ABI 7300 SDS. The relative expression of each mRNA was calculated by the comparative ΔΔCt method [56]. Endogenous β-actin mRNA levels were used for normalization of RNA expression.

### Methylation analyses -DNA extraction and bisulfite modification

MS-HRM [57] was used to detect DNA methylation of tumor suppressors (MT1G, NMES1, RRAD, SFRP1, SPARC and TFPI2). The method was based on PCR and the templates were treated with sodium bisulphate [58, 59]. Genomic DNA was extracted from uninfected (Mock) SiHa and CaSki cells and cells at different time points (0, 24, 48, 72 and 96 h) after infected with lentiviruses that expressed E6-, E7- and control shRNA using QiaAmp DNA Mini kit (Qiagen), according to the manufacturer's recommendation. The extracted DNA was quantified using a Nano Drop ND 2000c spectrophotometer (NanoDrop Thermo scientific). Genomic DNA was treated using a Epitect Bisulfite Kit (Qiagen) to transfer all unmethylated C to T, and to maintain methylated C as C, according to the protocol of the manufacturer. Modified DNA was maintained at −80°C until PCR amplification.

Methylation of the 6 genes mentioned above was carried out in viia@7 (ABI). The MS-HRM primers were designed by Sova et al [30], shown in Supplementary Table 2. All analyses were run according to the following conditions: 1 cycle of 95°C for 5 min, 50 cycles of 95°C for 10 s, 58°C for 30 s and 72°C for 10 s; followed by an HRM step of 95°C for 10 s and 50°C for 1 min, 65°C for 15 s, and continuous acquisition to 95°C at one acquisition per 0.2°C PCR was performed in a final volume of 25 μL, containing 12.5 μL of master mix (Qiagen), 10 pmol of each primer and 1 μL (almost 10 ng) of bisulfite modified DNA template. Each reaction was performed in triplicate. We analyzed 10% of the samples independently on separate occasions to verify the inter-assay variability and we observed a good reproducibility. Fig. 3 shows the melting profiles of the 6 genes analyzed. Fully methylated and unmethylated DNA from the EpiTect PCR Control DNA Set (Qiagen) were mixed to obtain the following ratios of methylation: 0%, 1%, 5%, 10%, 25%, 50% and 100%. Standard curves with known methylation ratios were included in each assay and were used to deduce the methylation ratio of each sample.

The methylation status of the candidate genes at the 5 time points were normalized relative to two normalization regions before and after the major fluorescence decrease reflected the melting of the PCR product. Output plots were in the form of normalized and sample melting curves. Based on the standard curves, HRM data were classified into different ranges of methylation by two independent observers.

### Immunoblot analysis

Protein extracts were prepared from uninfected, E6-, E7- and control shRNA-infected SiHa and CaSki cells at 48 and 96 h after infection as described previously [60, 61]. Total protein concentration was measured with an Enhanced BCA Protein Assay Kit (Beyotime, Jiangsu ). For electrophoresis, approximately 50 μg of protein per lane was resolved on 10% SDS-polyacrylamide gels and transferred to Immun-Blot PVDF membranes (Bio-Rad, Hercules, CA ). Membranes were incubated with a rabbit monoclonal antibodies against human DNMT1 and DNMT3A protein at 1:1000 dilution (CST, Boston, MA ); human DNMT3B and DNMT3L at 1:2000 dilution (Abcam, Cambridge, UK ); β-actin (CST, 1:1000 dilution) was used as internal control for protein loading and analysis. Bound antibody was detected by 20 × LumiGLO® Reagent and 20 × Peroxide (CST).

### Cell viability assay

Growth of E6-, E7-, control shRNA-infected and uninfected SiHa and CaSki cell lines was dected using the Cell CCK-8 (Dojindo Laboratories, Kumamoto, Japan) by the manufacturer. Briefly, cells were dispensed in triplicate into 96-well plates (5 × 10^3^ cells/well) and incubated overnight at 37°C. The cells were then treated with lentivirus. 10 μL of CCK-8 solution was added to each well of the plates at 9 different time points (0, 12, 24, 36, 48, 60, 72, 84 and 96 h), which were then incubated at 37°C for 1 h. The absorbance was then measured by multiskan Spectrum (Thermo Scientific) at a wavelength of 450 nm. Data were obtained from at three separate experiments done in triplicate.

### Apoptosis analysis

SiHa and CaSki cells were cultured in 6-cm dishes (1.5 × 10^4^ cells per dish). At 72 h after infection, floating cells in the medium and adherent cells were collected and combined. Apoptosis was quantified by flow cytometric analysis (BD FACSVerse™ Flow Cytometer) of Annexin V Apoptosis Detection Kit FITC (eBioscience, San Diego, CA) staining in conjunction with propidium iodide (PI). Annexin V (+) and PI (−) cells were considered to be in early apoptosis and the percentage of this kind of cells was calculated, least three separate experiments done in triplicate.

### Statistical analysis

All experiments were performed at least in triplicate and results were recorded. The SPSS16.0 software package was used for statistical analysis. Data were presented as mean ± standard error of the mean (SEM), Student's t test and One-way analysis of variance were used to analyze the significance between groups. P values of less than 0.05 were considered statistically significant.

## SUPPLEMENTARY MATERIAL FIGURE AND TABLES


