# Supplementary material for: E6 and E7 gene silencing results in decreased methylation of tumor suppressor genes and induces phenotype transformation of human cervical carcinoma cell lines
Source: Oncotarget. 2015 Jun 19;6(27):23930–43. doi: 10.18632/oncotarget.4525 (PMC4695162; doi:10.18632/oncotarget.4525)
Supplement: Supplementary file 1 [file oncotarget-06-23930-s001.pdf]

E6 and E7 gene silencing results in decreased methylation of tumor suppressor genes and induces phenotype transformation of human cervical carcinoma cell lines

Supplementary Material

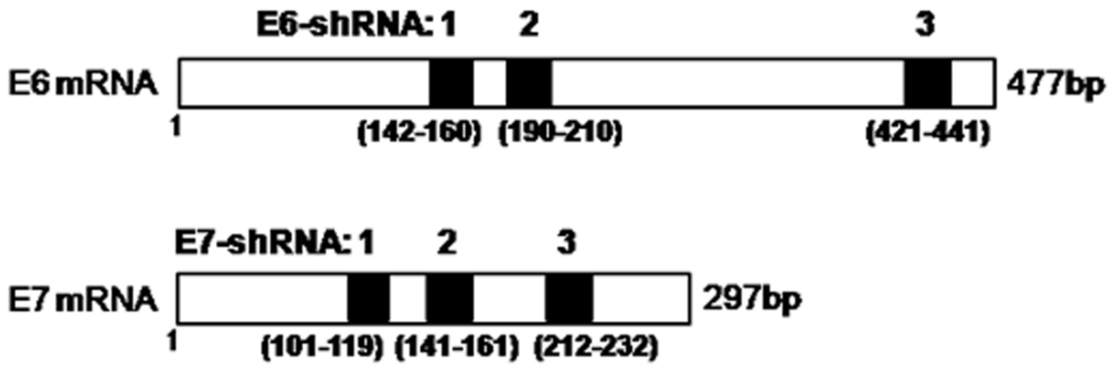

Supplementary Figure 1. Sketch map of the location of specific shRNAs on E6 and E7 genes of HPV16.

Supplementary Table 1. Sequences selected for generating shRNAs.

shRNA	Oligonucleotides sequence (5'-3')
E6-1	F: AATTCGAGGTATATGACTTTGCTTTTCAAGAGAAAGCAAAGTCATATACCTCTTTTTTG<br>R: GATCCAAAAAAGAGGTATATGACTTTGCTTTCTCTTGAAAAGCAAAGTCATATACCTCG
E6-2	F: AATTCGGGAATCCATATGCTGTATGTTTCAAGAGAACATACAGCATATGGATTCCCTTTTTTG<br>R: GATCCAAAAAAGGGAATCCATATGCTGTATGTTCTCTTGAAACATACAGCATATGGATTCCCG
E6-3	F: AATTCGGTCGATGTATGTCTTGTTGCTTCAAGAGAGCAACAAGACATACATCGACCTTTTTTG<br>R: GATCCAAAAAAGGTCGATGTATGTCTTGTTGCTCTCTTGAAGCAACAAGACATACATCGACCG
E7-1	F: AATTCAGGAGGATGAAATAGATGGTTCAAGAGACCATCTATTTCATCCTCCTTTTTTTG<br>R: GATCCAAAAAAAGGAGGATGAAATAGATGGTCTCTTGAACCATCTATTTCATCCTCCTG
E7-2	F: AATTCGGACAGAGCCCATTACAATATTTCAAGAGAATATTGTAATGGGCTCTGTCCTTTTTTG<br>R: GATCCAAAAAAGGACAGAGCCCATTACAATATTCTCTTGAAATATTGTAATGGGCTCTGTCCG
E7-3	F: AATTCGCACACACGTAGACATTCGTATTCAAGAGATACGAATGTCTACGTGTGTGCTTTTTTG<br>R:GATCCAAAAAAGCACACACGTAGACATTCGTATCTCTTGAATACGAATGTCTACGTGTGTGCG
Control	F: AATTCTTCTCCGAACGTGTCACGTTTCAAGAGAACGTGACACGTTCGGAGAATTTTTTG<br>R: GATCCAAAAAATTCTCCGAACGTGTCACGTTCTCTTGAAACGTGACACGTTCGGAGAAG

Supplementary Table 2. The primers for genes examined in our study.

Primer	Primer sequence (5'-3')	Useage
E6	F: CAGCAATACAACAAACCG<br>R: GCAACAAGACATACATCG	RT-PCR<br>Q-PCR
E7	F: CAGAGGAGGAGGATGAAATAG<br>R: AGGTCTTCCAAAGTACGAATG	RT-PCR<br>Q-PCR
DNMT1	F: CCACCATCACATCTCATTT<br>R: GGTCTAGCAACTCGTTCTC	Q-PCR
DNMT3A	F: GCGGAGGTCGGGAGAAC<br>R: CGATGGCTCCACCTGGC	Q-PCR
DNMT3B	F: AGACACCAGGCATCTCAAT<br>R: TCTTCGGCCTCTGATCTC	Q-PCR
DNMT3L	F: TCTGTTTGAGGGAGGGAT<br>R: GTAGCATCGGGTGCAATC	Q-PCR
SPARC	F: GCCCAGTTCATCACCATTTCCT<br>R: TCCCAGAGAACTGACAGTCCGT	Q-PCR
TFPI2	F: CTGTGCTCTGCCAATGTGACTC<br>R: TGCAATCCTCCCTGCTAACAA	Q-PCR
RRAD	F: ACTCAGTGACGGACAAGGG<br>R: CCTCATCCACCGAGACCT	Q-PCR
SFRP1	F: AAGTTTCGGTTCCTATGAGCCC<br>R: AAAATCGCCGTCTCTCTCAGG	Q-PCR
MT1G	F: TTGCAATGGACCCCAACTG<br>R: ACTTCTCCGATGCCCCTTT	Q-PCR
NMES1	F: TGACCAAATGACGAGCCCT<br>R: CAGAAGCATTTCCGCACACT	Q-PCR
β-actin	F: CCATCGTCCACCGCAAAT<br>R: GCTGTCACCTTCACCGTTCC	Q-PCR
SPARC	F: TGGAGGGGAGATAGATTTAGTTT<br>R: CAAAACTTCCCTCCCTTACAC	MS-HRM
TFPI2	F: TGGGGAGAGAAGTTTTTGGA<br>R: ATCAACCACCCCTCAAACTC	MS-HRM
RRAD	F: ATTTAGGGTTTTAGGTTGGATTGGT<br>R: ATCCCCTCACCTACTACCTCTACTTC	MS-HRM
SFRP1	F: GTTTTATTTTGGGGTTTGGAGGTTT<br>R: CAAACCAACAACTAAATACCCCTACTCA	MS-HRM
MT1G	F: ATGGTGTTTGGGAATTTGGT<br>R: AAACCTTACACTTAACCCATCTCCT	MS-HRM
NMES1	F: GTAGAATGTTTTGTAGGGGG<br>R: CTACAAATAAAAAACTCACCTAAAC	MS-HRM
